# Resource-Limited Management of Presumptive Pyoderma Gangrenosum in an Unsheltered Patient

**DOI:** 10.7759/cureus.21629

**Published:** 2022-01-26

**Authors:** Taha F Rasul, Megan Mathew, Jackson D Anderson, Daniel R Bergholz, Armen Henderson

**Affiliations:** 1 Department of Infectious Diseases, University of Miami Miller School of Medicine, Miami, USA; 2 Department of Dermatology, University of Miami Miller School of Medicine, Miami, USA; 3 Department of Internal Medicine, University of Miami Miller School of Medicine, Miami, USA; 4 Department of Allergy and Immunology, University of Miami Miller School of Medicine, Miami, USA; 5 Internal Medicine, University of Miami Hospital, Miami, USA

**Keywords:** pyoderma gangrenosum, medicine in resource-limited areas, immunosuppression, chronic inflammation of skin, delayed wound healing, homelessness, street medicine

## Abstract

Pyoderma gangrenosum (PG) is an ulcerating dermatosis associated with various chronic medical conditions. Its exact etiology is unknown but likely a function of inflammation and immune dysregulation. Treatment of PG generally follows a stepwise approach which involves extensive testing, biopsies, and potentially systemic therapy. However, patients with presumptive PG in an unsheltered homeless (USH) environment require a different approach, especially in a resource-limited setting. Our 65-year-old USH patient with an extensive medical history presented with an initial, irregular salmon-colored plaque measuring approximately 10 cm × 6 cm that eventually ulcerated with pain and purulent discharge. The consistent and judicious management of his wound in terms of gentle irrigation and appropriate dressing was performed over the course of seven months starting in April 2021. In November 2021, his wound margins shrunk by roughly 1 cm circumferentially, and the ulcer had scant serosanguinous discharge, a noticeable improvement from baseline. The previously impaired wound healing may have been due to pathergy, which was indirectly addressed by protective wound dressings. Management of chronic wounds and ulcers in patients otherwise lacking access to reliable care should avoid systemic immunosuppressants due to the inherently high-risk conditions on unsheltered streets.

## Introduction

Dermatological conditions are some of the most common complaints seen in unsheltered homeless (USH) individuals [1]. These are often chronic and untreated and usually present in later stages when there is significant morbidity present. As a result, many wounds encountered among the unsheltered have impaired healing and complications such as infection and sepsis [2].

Pyoderma gangrenosum (PG) is a neutrophilic dermatosis that manifests with painful, rapidly progressive, erythematous papules and/or pustules that can develop into deep, ulcerated lesions with central necrosis [3]. It is typically associated with inflammatory bowel diseases, autoimmunity, hematologic disorders, and trauma. The most common location of PG lesions is on the extensor surfaces of the extremities. There is typically a chronic disease course, and recurrence is not uncommon [4]. The mainstay of treatment is generally immunosuppression using drugs such as corticosteroids or cyclosporine. Some studies have reported that a stepwise approach can improve outcomes, especially when topical and systemic treatments are used simultaneously [5]. The stepwise approach consists of an initial clinical evaluation using biopsy results to confirm the initial suspicion and exclude the wide differential diagnoses, such as infections, malignancy, and vascular ulceration. Additionally, it involves identifying associated medical conditions underlying the change in the patient’s wound status. The second step is applying a local treatment using dressings, topical therapy, or a combination of the two. Wound dressings include hydrocolloid, antimicrobial, and alginate dressings. Topical corticosteroids are usually applied to attenuate the local inflammation. Additionally, larval therapy has had some therapeutic benefits in a few cases [6]. The third step in the escalation of treatment is systemic immunomodulation therapy such as intravenous corticosteroids, cyclosporine, and biologics. Refractory PG is treated with surgical therapy such as skin grafting, and in severe cases, amputation [7]. There is also an inherent risk for pathergy resulting from skin grafting. This is why systemic immunosuppression is often tested extensively before more invasive options.

Certain drug combinations have also been found to have an appropriate level of efficacy; although there is a variable response to treatment in general. For example, a trial of 112 patients in 2014 found similar efficacy in treatment between cyclosporine and prednisolone, even when used simultaneously [8]. However, the study also found that fewer than half of the ulcers healed after prolonged treatment, and the speed of the response did not differ significantly. More superficial PG lesions can be managed with topical therapy as an adjunct to systemic immunosuppression. The improvement in inflammation and pathergy can be measured as a function of the ulcer edges becoming more homogenous with the surrounding skin [9]. Even so, the complete resolution of pathogenic inflammation is not a reliable indicator of wound healing. Approximately 50% of PG patients achieve remission after six months of immunosuppression. Relapses are estimated to occur in 30-60% of patients who initially responded well to therapy [10].

For individuals with very limited resources and access to care, such as the USH, the presence of PG can be rather debilitating. Although no studies have been conducted into the prognosis of PG in this population, it is likely that the lack of longitudinal treatment makes their rates of resolution lower and relapse higher than that of the general population. For individuals treating USH patients with PG, the stepwise approach becomes quite important. This is because individuals living on the street have a generally increased risk of contracting illnesses such as tuberculosis. Therefore, systemic immunosuppression may lead to a sharp deterioration in their overall health condition. Additionally, the cost of such medications without insurance may become unsustainable to maintain. This paper describes a case of PG in a USH patient and explores the treatments provided, improvements that could be made, and the general approach to managing complicated lesions in such high-risk patients.

## Case presentation

Mr. P. is a 65-year-old USH patient with a medical history including chronic wounds, hepatitis C, cirrhosis, right eye blindness, presumptive end-stage liver disease, and major depressive disorder who was seen for wound care by the Miami Street Medicine (MSM) team between April and November 2021. Mr. P has a history of chronic, non-healing wounds and is often unable to dress them appropriately. Most of his medical history is unknown, but he described having chronic wounds on his shins for at least a year. Additionally, his history includes multiple hospitalizations secondary to infection. His exposed, non-healing wounds were likely a consistent nidus for infection. The environment on the streets where he lives is also untidy and littered with significant amounts of dirt, used syringes, and garbage. His lack of access to timely laundry also meant that the only covering for the wound was his pants, which had not been washed in months.

**Figure 1:**
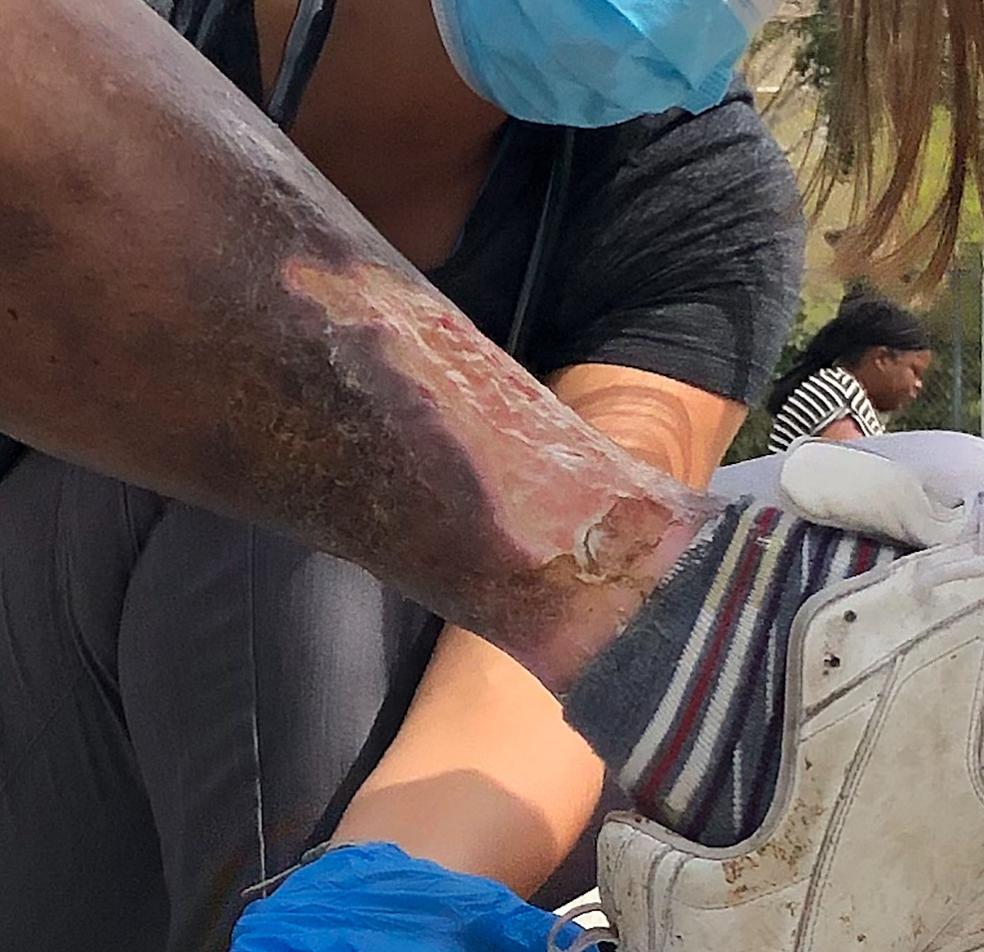
Initial wound on the distal left shin. Notice the salmon-colored plaque nature which would eventually ulcerate.

The initial evaluation of Mr. P. addressed his most immediate status and vitals. He was recommended to go to the emergency department for evaluation of his multiple conditions; however, he refused, likely a consequence of the distrust that many USH patients have for institutions, including the medical system. His chief concern was the lesion on his left leg. It initially began as a large, pink plaque on his anterior shin, as seen by the street team (Figure 1). It should be noted that the patient was a poor historian and was not sure about the chronology of his lesion. Approximately two weeks later, he presented with ulceration of the same lesion which was now warm, painful, and exudative.

**Figure 2:**
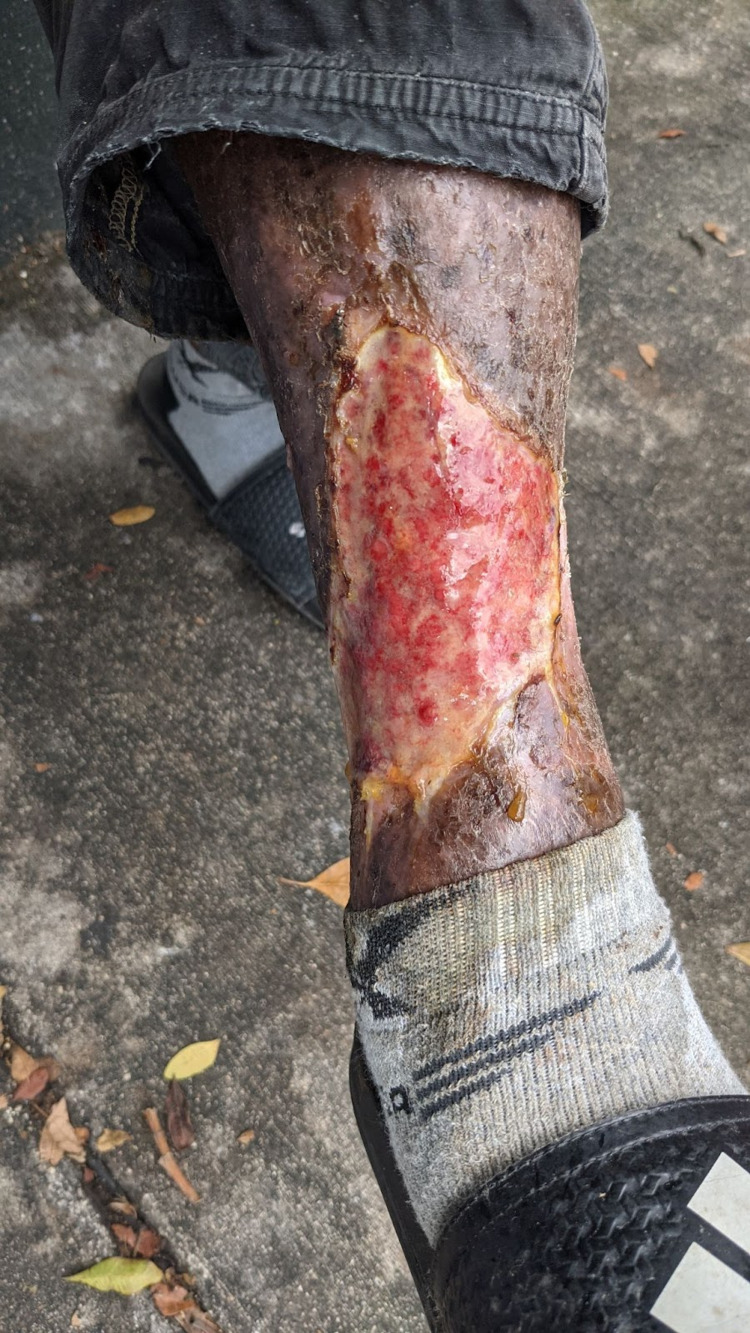
Ulcerated wound one week later. The wound edges are sharply demarcated and the wound itself had notable purulent discharge. Our patient had been covering the wound with his pant leg.

Although he refused treatment initially, during the following visit one week later, he agreed to the debridement and bandaging of his wound. He continued to vehemently refuse any emergency department visit or ambulance transport. On examination, his wound appeared to be a 10 cm × 6 cm superficial ulcerated lesion with raised, defined borders with necrotic tissue around the periphery. Insects including flies and maggots were seen around the wound. There was a moderate amount of foul and purulent drainage from the wound (Figure 2).

**Figure 3:**
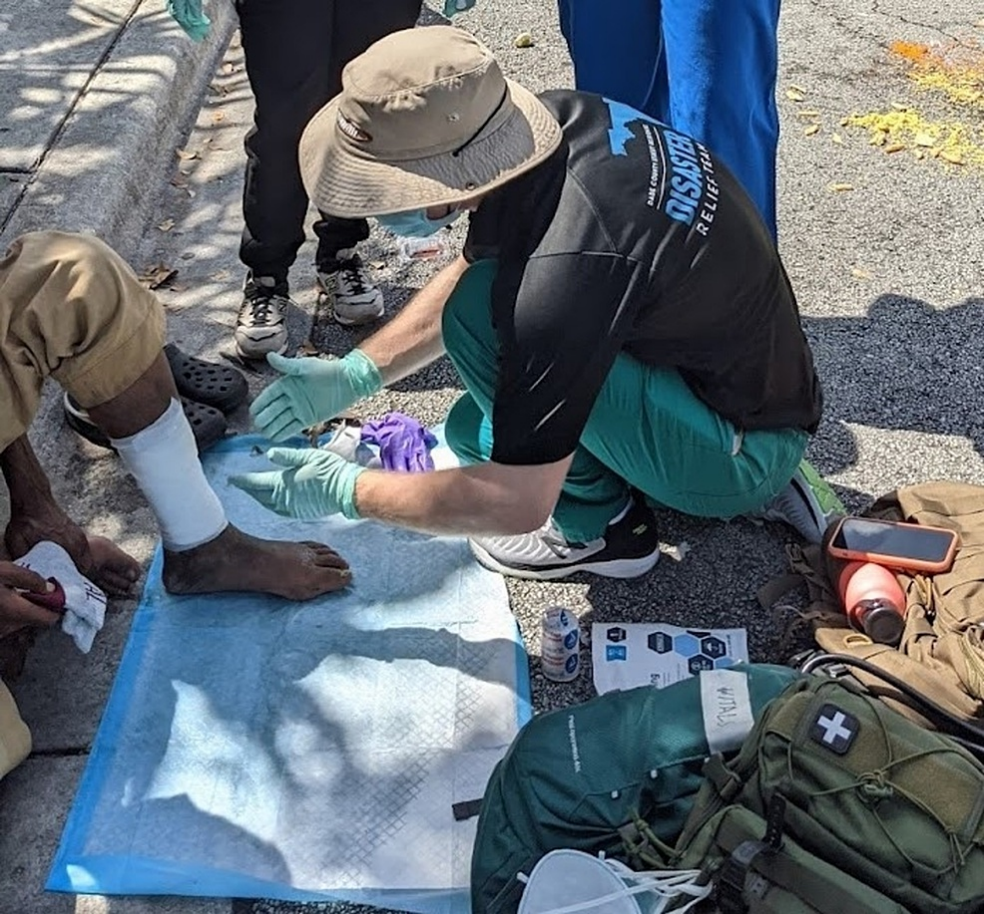
Example of wound care provided in August 2021 by Street Medicine teams in a low-resource setting. Our patient’s wound was covered in a non-adhesive foam dressing and wrapped with supportive gauze.

His ulcerated wound was gently irrigated with a sterile normal saline solution. A non-adhesive 15 cm × 15 cm foam dressing was applied to the lesion and buttressed with two separate layers of gauze followed by a cotton elastic bandage (Figure 3). This created a closed seal which isolated the wound from the outside environment and particularly blocked insects from further irritating the lesion. He was also provided wound-care supplies to change his dressing after a few days. Due to his general mistrust of the medical system, it was not possible to further evaluate the lesion with a confirmatory biopsy.

**Figure 4:**
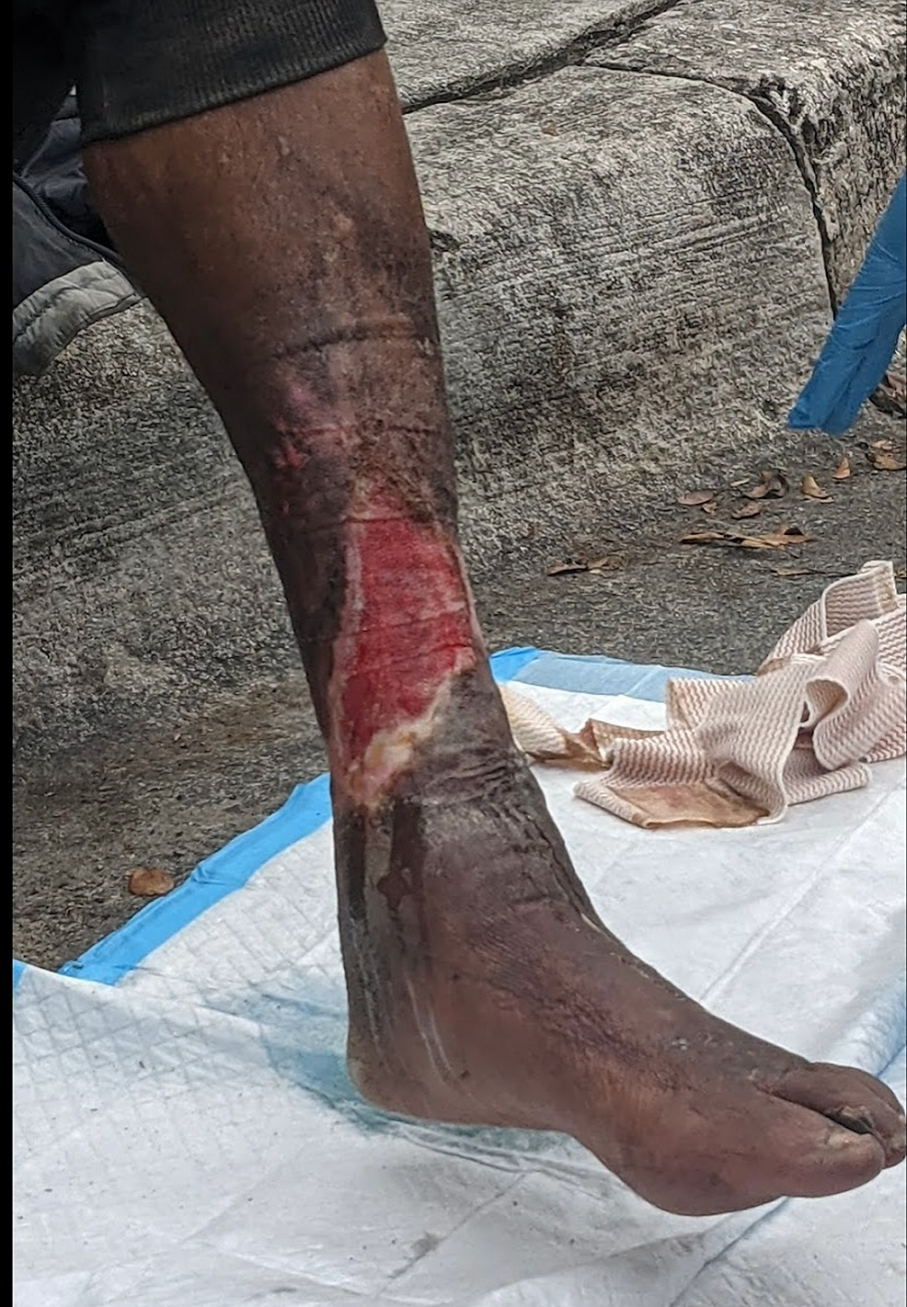
Partially resolved lesion noted after seven months of regular dressing changes and follow-up. Pink granulation tissue was noted in the wound and the margins were less raised. The discharge was noted to be serosanguinous.

Over the next seven months, he was regularly seen at the Miami Street Medicine clinic where his wound was cleaned and dressed appropriately. By early November 2021, there was a noticeable improvement in the size and character of the lesion (Figure 4). The size of the wound decreased by approximately 1 cm, the wound edges were well-defined, and healthy granulation tissue was seen at the wound base. There was mild serous drainage. The patient also reported a subjective improvement in pain.

## Discussion

Our 65-year-old patient with an extensive, mostly unknown, medical history was treated for his presumptive PG lesion. This occurred over the course of seven months at a student-run mobile clinic in the Miami Medical District. His treatment was mostly a function of limited resources, both from the patient and provider ends. The lack of insurance and hesitancy to be evaluated in a formal clinical setting also meant that the majority of the care and wound healing process occurred on the street. Mr. P notably suffered from many comorbidities due to living in an unsheltered environment. First, his overall health was not optimal, and he suffered from malnutrition and food insecurity. He was often unsure about where and when he would acquire his next meal. His clothes were unkempt, and he would often go months at a time wearing the same socks and undergarments. His living environment was unsafe both in terms of health and personal safety. As mentioned before, his wounds were previously colonized with insects and maggots. Although larval therapy is sometimes employed in the treatment of PG, it is done in a clinically controlled environment unlike the unsheltered living space of our patient. Therefore, if his underlying medical conditions were the igniting cause of his ulcerations, his living conditions likely exacerbated the delayed resolution of his PG.

Consistent wound care by the street medicine team likely allowed for wound healing to occur in the absence of aggravating factors, such as those found in an unsheltered environment. Local or systemic corticosteroids and immunomodulators were not used in treatment due to a lack of appropriate baseline laboratory results, in addition to financial constraints, lack of reliable follow-up, and nutritional status. Additionally, the high propensity of infection, particularly due to close contact with other USH patients, meant that the initial course of action was conservative management. The first step of PG management has generally been confirmatory testing, usually in the form of a lesion biopsy which also excludes various differential diagnoses [11]. Other conditions that could cause a similar, large, ulcerating lesion include infectious, traumatic, and neoplastic etiologies. Certain atypical mycobacterial infections such as tuberculous gummas can have similar presentations but would persist in the absence of antibiotic treatment [12]. Neoplastic ulcerations such as squamous cell carcinoma (Marjolin’s ulcers) arise in areas of repeated trauma and inflammation. They usually persist and expand if left untreated. Although we were unable to obtain a biopsy of the lesion, the general course of the disease with conservative management suggests a very atypical picture of PG. The ulcer did not heal completely by the time of our latest evaluation in November, but there was noticeable improvement in the size, depth, drainage, and pain.

The management of this case of presumed PG in a resource-scarce setting revolved around dressings and topical therapy, with specialized care taken to ensure consistency. It is likely that the longitudinal care provided to Mr. P was a major factor in the improvement of his wound. One verified part of his medical history is his chronic hepatitis C, which has been noted to be an inciting factor for conditions such as PG, albeit not as common as inflammatory bowel disease. Although there is not a well-established link between PG and hepatitis C, it has been found that PG generally arises in association with various inflammatory, autoimmune, and hematologic disorders. This suggests that systemic inflammation or some other form of longstanding dysfunction led to the formation of his lesion.

One notable consideration that is required with PG is pathergy [13]. Repetitive trauma can incite or exacerbate PG, and hence, care must be taken to avoid trauma or disruption of the wound. This could be a reason for our patient’s gradual improvement because the irrigation was done in a gentle manner which did not disturb the overall wound character. The dressing’s effect on insulating the wound from external trauma may have allowed healing on an ulcer that was otherwise stable or deteriorating [14,15]. Therefore, this presumptive case of PG showed noticeable improvement with judicious wound irrigation, non-adhesive foam dressing buttressed with additional gauze layers, and regular follow-up. Through Mr. P’s case, we hope to highlight the necessity and challenges of providing wound care to those who would otherwise be unable or unwilling to seek help from the medical system. Even though our patient did possess the mental capacity to refuse hospital treatment, situations with a lack of capacity coupled with life-threatening infections or wounds may be managed by contacting emergency medical services. Further, with chronic wounds being a major complaint among USH patients, this case report points out the impact that regular follow-up and effective wound care can have on improving outcomes for USH patients.

## Conclusions

Wound care is one of the most common concerns in the USH population due to the numerous risk factors that both predispose this population to wounds and hinder optimal wound healing. The wounds that these patients suffer from exist in the unique setting of homelessness and often other comorbidities including intravenous drug use, human immunodeficiency virus/hepatitis C virus infection, and chronic diseases such as diabetes, autoimmune disorders, and mental health conditions. As a result, homeless patients with chronic wounds require specialized care that may not follow protocols used in a clinic setting. There are few updated clinical guidelines and minimal literature describing the medical treatment of complex dermatologic conditions on the streets seen in the USH.

PG is a neutrophilic dermatosis that can have varying presentations. Although medical management generally follows a stepwise approach, certain treatment options may not be feasible in situations with limited resources such as on the streets. Diagnosis of PG is traditionally confirmed through biopsy; however, treatment should not be delayed if history and clinical features are consistent with PG and biopsy is not possible. Conservative, yet consistent, wound care and longitudinal follow-up had an appreciable effect on several metrics of wound healing in our patient’s chronic lower extremity ulcer, suspected to be PG. For our patient living on the street, systemic immunosuppression may not have been an appropriate treatment modality due to the inherently higher risk of infection and communicable disease transmission. Providers caring for patients in street settings should adapt their treatment practices to best suit the living conditions of USH patients. This should also include a holistic assessment of nutritional status which can be a major barrier for complete wound resolution, as well as providing nutritional support in the form of multivitamins and soup kitchen referrals.
